# Regulating electron transportation by tungsten oxide nanocapacitors for enhanced radiation therapy

**DOI:** 10.1186/s12951-023-01962-8

**Published:** 2023-06-29

**Authors:** Hongbo Gao, Li Sun, Dalong Ni, Libo Zhang, Han Wang, Wenbo Bu, Jinjin Li, Qianwen Shen, Ya Wang, Yanyan Liu, Xiangpeng Zheng

**Affiliations:** 1 Department of Radiation Oncology, Shanghai Huadong Hospital, Fudan University, Shanghai, 200040 China; 2 Department of Orthopaedics, Shanghai Key Laboratory for Prevention and Treatment of Bone and Joint Diseases, Shanghai Institute of Traumatology and Orthopaedics, Ruijin Hospital, Shanghai Jiao Tong University School of Medicine, Shanghai, 200025 China; 3 Department of Radiology, The First Affiliated Hospital of Soochow University, Suzhou, 215006 China; 4 Department of Material Science and State Key Laboratory of Molecular Engineering of Polymers, Fudan University, Shanghai, 200433 China; 5 Shanghai Key Laboratory of Green Chemistry and Chemical Processes, School of Chemistry and Molecular Engineering, East China Normal University, Shanghai, 200062 China

**Keywords:** Tungsten oxide, Cancer, Radiotherapy, Nanotechnology, Pseudocapacitor

## Abstract

In the process of radiation therapy (RT), the cytotoxic effects of excited electrons generated from water radiolysis tend to be underestimated due to multiple biochemical factors, particularly the recombination between electrons and hydroxyl radicals (·OH). To take better advantage of radiolytic electrons, we constructed WO_3_ nanocapacitors that reversibly charge and discharge electrons to regulate electron transportation and utilization. During radiolysis, WO_3_ nanocapacitors could contain the generated electrons that block electron-·OH recombination and contribute to the yield of ·OH at a high level. These contained electrons could be discharged from WO_3_ nanocapacitors after radiolysis, resulting in the consumption of cytosolic NAD^+^ and impairment of NAD^+^-dependent DNA repair. Overall, this strategy of nanocapacitor-based radiosensitization improves the radiotherapeutic effects by increasing the utilization of radiolytic electrons and ·OH, warranting further validation in multiple tumour models and preclinical experiments.

**Supplementary Information:**

The online version contains supplementary material available at 10.1186/s12951-023-01962-8.

## Introduction

Radiation therapy (RT) is one of the most effective cytotoxic therapies for the treatment of malignant tumours, and approximately 60% of patients receive radiotherapy in different treatment stages despite advances in many other treatment modalities [1–3]. Basically, the interaction between X-rays and genetic materials (mainly DNA molecules) contributes to cancer cell death. Radiation photons can generate direct damage to DNA or react with adjacent water molecules to produce highly reactive hydroxyl radicals (·OH) followed by DNA damage (the indirect mechanism) [4–8]. It has been recognized that the majority of X-ray damage to DNA in mammalian cells is caused by ·OH. In addition to ·OH, free electrons are also produced during the radiolysis of H_2_O. Despite potential contributions to DNA damage, these free electrons have negative impacts on the collective radiation effects due to recombination with ·OH and resultant neutralization of ·OH [9–12]. Hence, it is theorized to interfere with or block the recombination process of electrons and ·OH to take better advantage of both electrons and ·OH to improve the clinical outcome of radiotherapy.

The DNA damage response (DDR) is the cellular self-defence response to DNA damage with attempts to recover the integrity of DNA molecules, which apparently compromises radiotherapeutic efficacy [13–15]. As the most common type of DNA damage, DNA single-strand breaks (SSBs) tend to be readily repaired by the PARP-1-mediated signalling pathway, and NAD^+^ exerts a critical role in the process of activating this pathway [16–18]. Reducing NAD^+^ to NADH can effectively suppress the repair of DNA SSBs, and unrepaired DNA SSBs can transform into DNA double-strand breaks (DSBs) at replication forks, which may be irreparable or difficult to repair and ultimately fatal to cells [19]. Thus, it would be ideal to instruct radiolytic electrons to react with NAD^+^, which not only separates electrons and ·OH to increase the utilization efficiency of ·OH but also simultaneously reduces the intracellular NAD^+^ content to suppress DNA SSB repair. However, considering the supershort lifetime of radiolytic electrons (typically at the microsecond level), the reaction window between NAD^+^ and electrons is extremely narrow under natural conditions [19, 20]. How to transfer radiolytic electrons to NAD^+^ remains a significant issue to be solved.

In light of their unique performance in electron storage and release, supercapacitors have been widely studied in renewable energy fields for decades [21, 22]. Among various supercapacitors, WO_3_, RuO_3_ and MnO_2_ have received much attention due to their high capacitance and energy density [23–27]. During the pseudocapacitance reaction, metal ions reversibly absorb and release electrons for time-space resolved regulation of electrons [28, 29]. This process suggests that pseudocapacitors may have potential medical use by regulating electron transportation by reversibly storing and releasing X-ray-induced electrons to extend their lifetime to overcome the above mentioned issues related to radiolytic electrons.

**Fig. 1:**
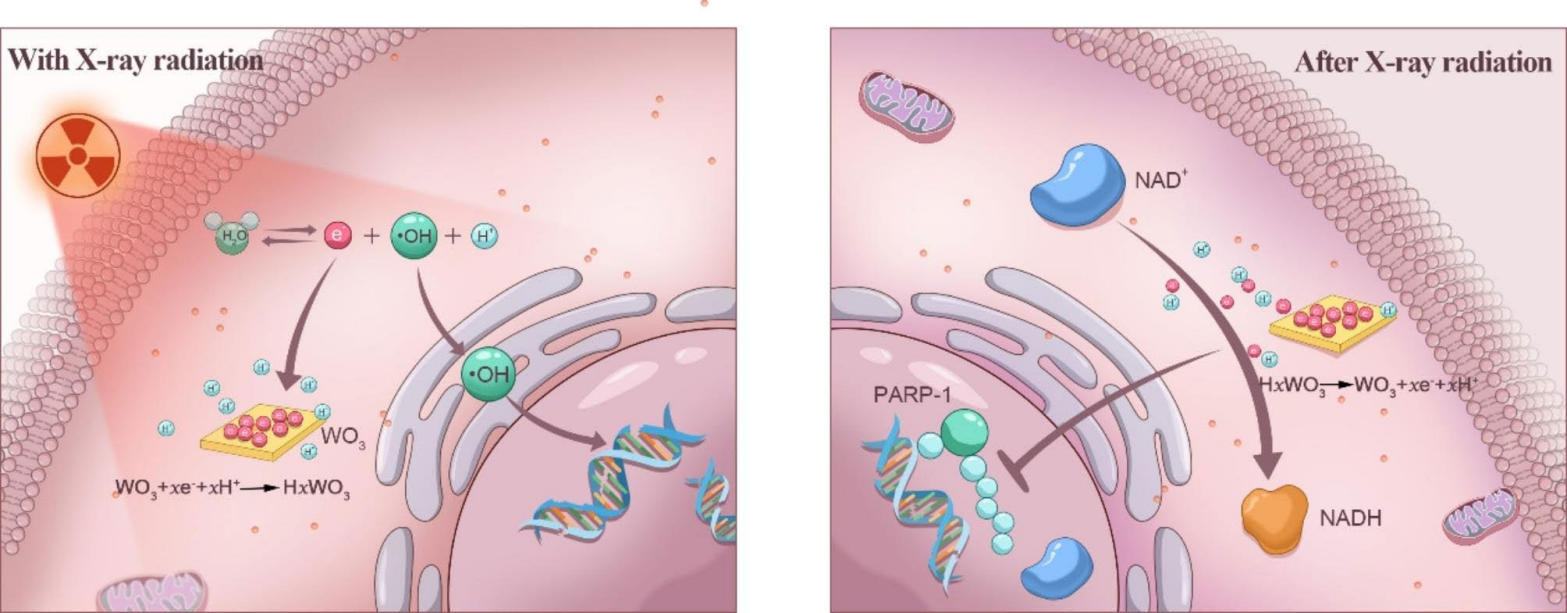
Illustration of the mechanism that regulating electron transportation by WO_3_ nanocapacitors for enhanced radiation therapy

Herein, we report the experimental results of WO_3_ nanocapacitors as electron regulators for radiosensitization. As demonstrated in Fig. 1, during the process of radiolysis, WO_3_ nanocapacitors accepts radiolytic electrons (charging), which reduces the probability of recombination between ·OH and electrons to maintain cytosolic/nucleic ·OH at a high level. Subsequently, these electrons are discharged from nanocapacitors to react with endogenous NAD^+^, potentially resulting in NAD^+^-dependent reduced PARP-1-mediated DNA SSB repair. Collectively, the employment of WO_3_ nanocapacitors enhances radiation-induced DNA damage and radiotherapeutic effects.

## Methods

### Reagents

Na_2_WO_4_·2H_2_O, citric acid and glucose were purchased from Adamas-beta. Hydrochloric acid (37%) was obtained from Sinopharm. LuCl_3_·6H_2_O was purchased from Sigma‒Aldrich. Rhodamine B (RhB) was purchased from TCI. The aminophenyl fluorescein (APF) probe was purchased from AAT Bioquest. Phosphate buffered solution (PBS), Dulbecco’s modified Eagle medium (DMEM) and foetal bovine serum (FBS) were obtained from Gibco. Cell counting kit-8 (CCK-8), histone H_2_AX rabbit polyclonal antibody, DAPI staining kit, annexin V-FITC apoptosis detection kit, haematoxylin and eosin (H&E) staining kit, NADH/NAD^+^ assay kit with WST-8 and TUNEL apoptosis assay kit were purchased from Beyotime.

### Cells and animals

Lewis cancer cells (mouse lung cancer cells), RAW264.7 and H9C2 cells were purchased from Shanghai Institute of Biochemistry and Cell Biology, Chinese Academy of Sciences. Kunming mice and Balb/c nude mice were purchased from Shanghai SLAC Laboratory Animal Co. Ltd. These mice were raised in the Laboratory Animal Center of Fudan University. All in vivo experiments were approved by the Animal Care Committee of Laboratory Animals of Fudan University.

### Experimental apparatus

A transmission electron microscope (TEM) graph was obtained from FEI Tecnai G2 F30. X-ray diffraction (XRD) was measured by Rigaku D/MAX-2250 V. The UV‒Vis absorption spectrum was measured by Shimadzu UV-3600 Plus. Inductively coupled plasma‒optical emission spectrometry (ICP‒OES) was measured by Agilent Technologies 5100. The electrochemical workstation was CHI660E. Confocal laser scanning microscopy was carried out on a Nikon A1^+^R-980. The fluorescence microplate system was TECAN SPARK. The fluorescence spectrometer was an Edinburgh Instruments FLS 980. Dynamic light scattering (DLS) was measured by a Malvern ZEN1600. Deionized (DI) water was obtained from ELGA CENTRA. The flow cytometer was a Beckman CytoFlex S. Radiation therapy was carried out by a Varian Clinac 21EX (Trilogy), which was applied in the clinic at Huadong Hospital affiliated with Fudan University. X-rays (6 MeV) were used for all experiments, and the dose rate was 5 Gy/min.

### Synthesis of WO_3_ nanocapacitors

60 mL deionized water, 3 mmol citric acid, 2 mmol Na_2_WO_4_·2H_2_O and 10 mmol glucose were mixed and stirred until the solution was clear. Then, this solution was poured into a 100 mL hydrothermal synthesis reactor and stirred. After the slow and dropwise addition of 3.6 mL hydrochloric acid, this mixture was stirred for another 30 min. Then, this reactor was placed in a heating box at 120 ℃ for 20 h to obtain WO_3 − x_. After the reaction, the supernatant in the reactor was discarded. The obtained WO_3 − x_ at the bottom was rinsed with deionized water and collected via high-speed centrifugation three times. To eliminate oxygen vacancies, WO_3 − x_ was calcined at 500 ℃ for 10 h. Finally, the obtained pale yellow powder was a WO_3_ nanocapacitor.

### Electrochemical measurements

The electrolyte was 0.5 mol/L aqueous H_2_SO_4_. The reference electrode was a Ag/AgCl electrode. The counter electrode was a Pt electrode. The current collector was carbon cloth, and the area was 1 cm^2^. Each carbon cloth contained 4 mg WO_3_. For cyclic voltammetry (CV), the scan rates were 5, 10, 20, 40, 60, 80 and 100 mV/s. For galvanostatic charge‒discharge (GCD) measurements, the current densities were 0.5, 2.0, 5.0 and 10.0 A/g, respectively. Cycle performance was measured by GCD at 5.0 A/g. The applied potential window ranged from − 0.65 to 0.05 V. The frequency range of the electrochemical impedance spectra (EIS) was 0.01 to 10^5^ Hz with an amplitude of 10 mV. The specific capacitance of WO_3_ was calculated by GCD.

### Detection of •OH in solutions and in vitro

The yield of •OH was detected by the degradation of RhB. A single RhB solution (10 mg/L), WO_3_ (total mass 231.85 mg/L, W 1 mmol/L) solution containing RhB (10 mg/L), Na_2_WO_4_·2H_2_O (total mass 329.86 mg/L, W 1 mmol/L) solution containing RhB (10 mg/L), and LuCl_3_·6H_2_O (total mass 389.42 mg/L, Lu 1 mmol/L) solution containing RhB (10 mg/L) were prepared. Then, these solutions were transferred into 96-well plates (100 µL/well) and irradiated with 0 Gy, 5 Gy, 10 Gy, 15 Gy, 20 and 25 Gy X-rays (6 MeV, 5 Gy/min). The absorbance of RhB at 564 nm was measured via a microplate spectrophotometer. The decrease in the absorbance of RhB represented the yield of •OH. For the in vitro experiments, Lewis cells (1 × 10^4^ cells/well) were seeded in confocal dishes and incubated with RPMI-1640 medium, Na_2_WO_3_·2H_2_O (28.5 µg/mL) and WO_3_ nanocapacitors (20 µg/mL) for 12 h. Then, the culture medium was replaced with culture medium containing HPF (5 µmol/L) and incubated for 0.5 h. Afterwards, these dishes were irradiated with 4 Gy X-rays. Finally, the fluorescence of the HPF probe was observed by confocal fluorescence microscopy.

### Computer simulation

We employed first principles to perform density functional theory (DFT) calculations within the generalized gradient approximation (GGA) using the Perdew-Burke-Ernzerhof (PBE) formulation. We have chosen the projected augmented wave (PAW) potentials to describe the ionic cores and take valence electrons into account using a plane wave basis set with a kinetic energy cut-off of 500 eV. Partial occupancies of the Kohn − Sham orbitals were allowed using the Gaussian smearing method and a width of 0.05 eV. The electronic energy was considered self-consistent when the energy change was smaller than 10^− 6^ eV. A geometry optimization was considered convergent when the energy change was smaller than 0.02 eV/Å. In the process of simulating the crystal surface calculation, to avoid repetition for surface interaction, the vacuum spacing in the discontinuous direction was 15 Å for the (110) slab. In all the calculations, we use 6 × 6 × 4 for the Monkhorst-Pack k-point for the periodic interface model. We created W^5+^ by building an O vacancy model with a low concentration in the WO_3_ structure.

### Stability of WO_3_ nanocapacitors

WO_3_ nanocapacitors were dispersed in RPMI-1640 medium (concentration: 200 µg/mL). After 1 day, 5 days and 10 days, we characterized the hydrodynamic radius of each sample. For detecting the release of W atoms, we measured the concentration of W of supernatant by ICP-OES, which was collected via high-speed centrifugation.

### Cell viability

Lewis cells (1 × 10^4^ cells/well) were seeded in 96-well plates and cultured at 37 °C for 24 h. Then, the culture medium was replaced with fresh RPMI-1640 medium containing WO_3_ nanocapacitors at various concentrations (0, 6.25, 12.5, 25, 50, 100, 200, 400 µg/mL) and cultured for another 24 h. Then, the medium was discarded, and the cells were washed with PBS. Then, 100 µL of medium containing 10 µL of CCK-8 solution was added and coincubated for 2 h. Finally, the absorbance was measured by a microplate reader at 450 nm. The in vitro cytotoxicity of WO_3_ nanocapacitors to normal cells (RAW264.7 and H9C2 cells) was also assessed using a similar protocol.

### Detection of NAD^+^ in vitro

The NAD^+^ content in vitro was measured using WST-8 assays. Lewis cells were seeded in six-well plates and incubated with RPMI-1640 medium, Na_2_WO_3_·2H_2_O (28.5 µg/mL) and WO_3_ nanocapacitors (20 µg/mL) for 12 h. Then, the cells were treated with 4 Gy X-rays. After 0.5 h, the NAD^+^/NADH ratio was measured with an NAD^+^/NADH assay kit. The absorbance of solutions at 450 nm was measured by a microplate spectrophotometer, which presented the content of NAD+/NADH.

### Detection of PAR in vitro

Lewis cells (1 × 10^4^ cells/well) were seeded in confocal dishes and cultured for 24 h. Then, the cells were incubated with RPMI-1640 medium, Na_2_WO_3_·2H_2_O (28.5 µg/mL) and WO_3_ nanocapacitors (20 µg/mL) for another 24 h. Next, these cells were irradiated with 4 Gy X-rays. After 0.5 h, the cells were fixed with 4% paraformaldehyde for 10 min and washed with PBS three times. Then, 0.2% Triton X-100 was added to penetrate cells for 10 min. Afterwards, the cells were blocked with 1% BSA for 1 h. After incubation with PAR antibody for 12 h at 4 °C, the cells were treated with anti-rabbit IgG (H + L) and F(ab’)2 Fragment (Alexa Fluor®594 Conjugate) for 1 h and then stained with DAPI for 15 min. Finally, the fluorescence of PAR was observed using a confocal fluorescence microscope.

### Western blot analysis

Lewis cells (1 × 10^5^ cells/well) were seeded in 6-well plates for 24 h. Then, the cells were incubated with RPMI-1640 medium, Na_2_WO_3_·2H_2_O (28.5 µg/mL) and WO_3_ nanocapacitors (20 µg/mL) for another 24 h. Next, these cells were irradiated with 4 Gy X-rays and washed with PBS three times. The cells were all lysed with RIPA buffer (Absin) supplemented with PMSF buffer (Absin). Protein concentration was tested by using a BCA protein assay kit (Beyotime Biotechnology). Equal amounts of proteins were separated by sodium dodecyl sulfate‒polyacrylamide gel electrophoresis (SDS‒PAGE) and then transferred to nitrocellulose (NC) membranes (Pall Corp.) and incubated overnight with primary antibodies at 4 °C followed by blocking with bovine serum albumin (BSA) (5%, v/v). The primary antibodies included Anti-PARP1 (rabbit, Abcam, ab32138, 1:1000), Poly/Mono-ADP Ribose (rabbit, CST, 83,732 S, 1:1000) and Gapdh (rabbit, Abways, AB0037, 1:5000). The membranes were probed with relevant secondary antibodies and scanned by 492 Odyssey instruments (LI-COR).

### Detection of DNA DSBs in vitro

Lewis cells (1 × 10^4^ cells/well) were seeded in confocal dishes and cultured for 24 h. Then, the cells were incubated with RPMI-1640 medium, Na_2_WO_3_·2H_2_O (28.5 µg/mL) and WO_3_ nanocapacitors (20 µg/mL) for another 24 h. Next, these cells were irradiated with 4 Gy X-rays. Subsequently, the cells were fixed with 4% paraformaldehyde for 10 min and washed with PBS three times. Then, 0.2% Triton X-100 was added to penetrate cells for 10 min. Afterwards, the cells were blocked with 1% BSA for 1 h. After incubation with γ-H_2_AX antibody for 12 h at 4 °C, the cells were treated with anti-rabbit IgG (H + L), F(ab’)2 fragment (Alexa Fluor®488 Conjugate) for 1 h and then stained with DAPI for 15 min. Finally, the fluorescence of γ-H_2_AX was observed using a confocal fluorescence microscope.

### Flow cytometry assay

Lewis cells (1 × 10^5^ cells/well) were seeded in 6-well plates for 24 h. Next, these cells were incubated with RPMI-1640 medium, Na_2_WO_3_·2H_2_O (28.5 µg/mL) and WO_3_ nanocapacitors (20 µg/mL) for another 24 h. Then, the cells were irradiated with 4 Gy X-rays and washed with PBS three times. After incubation with RPMI-1640 for 24 h, the cells were stained with Annexin V-FITC and PI. Finally, cell apoptosis was measured by flow cytometry.

### Cell clone formation assay

Lewis cells were seeded in 6-well plates (200, 200, 400, and 800 cells per well) for 24 h. Next, the cells were incubated with RPMI-1640 medium, Na_2_WO_3_·2H_2_O (28.5 µg/mL) and WO_3_ nanocapacitors (20 µg/mL) for another 24 h and subsequently exposed to 0 Gy, 2 Gy, 4 Gy, and 6 Gy X-rays. After culturing for 10 days, the cells were fixed with methyl alcohol and stained with haematoxylin and eosin. The number of colonies was counted by ImageJ.

### In vivo biocompatibility assay

To detect the biocompatibility of WO_3_ nanocapacitors, blood parameter analysis and standard H&E staining were conducted using Kunming mice (7 weeks, female). After injection with WO_3_ nanocapacitors (50 mg/kg) via the tail vein, the main tissues (heart, liver, spleen, lung and kidney) of Kunming mice were obtained for H&E staining, and blood was harvested for routine blood tests and biochemical examination at 2 days and 30 days postinjection. The control group received intravenous PBS only and was sacrificed at 2 days and 30 days for the same assays.

### In vivo blood terminal half-life and biodistribution of WO_3_ nanocapacitors

To assay the blood terminal half-life of WO_3_ nanocapacitors in vivo, three Kunming mice (8 weeks, female) were injected with 100 µL of WO_3_ nanocapacitors (50 mg/kg) via the tail vein. Then, 10 µL blood was obtained from the tail vein at various time points of 5 min, 10 min, 0.5 h, 1 h, 2 h, 4 h, 8 h, 12 and 24 h postinjection. Next, the blood samples were diluted with 990 µL of deionized water containing 10 mM ethylenediaminetetraacetic acid disodium salt as a blood anticoagulant. The concentration of WO_3_ (W element) was measured by ICP‒OES. The biodistribution of WO_3_ nanocapacitors in vivo was also determined using Kunming mice by intravenous injection of 100 µL of WO_3_ nanocapacitors (50 mg/kg). After 24 h, the major organs (heart, liver, spleen, lung and kidney) were harvested and dissolved in aqua regia. Finally, the W element concentrations were detected by ICP‒OES.

### In vivo radiation therapy

To establish the xenograft tumour model, first, the xenograft tumour model was established by subcutaneously injecting Lewis cells (1 × 10^6^ cells) into the flanks of Balb/c nude mice (7 weeks, female). When the tumour volume reached approximately 80–100 cm^3^, the mice were randomly divided into four groups: (i) control, (ii) WO_3_, (iii) Na_2_WO_3,_ (IV) control + X-ray, (v) Na_2_WO_3_ + X-ray, and (vi) WO_3_ + X-ray. PBS (10 µL), Na_2_WO_3_·2H_2_O (1.4 mg, 10 µL) and WO_3_ nanocapacitors (1 mg, 10 µL) were injected into tumours directly. After 12 h, the tumours of groups (iii, iv) were irradiated with 6 Gy X-rays. After 48 h, H&E and TUNEL staining of the tumour tissues was performed using commercially available kits. The body weight and tumour volume of the mice were measured every 3 days.

## Results and discussion

### Preparation and characterization of WO_3_ nanocapacitors

**Fig. 2:**
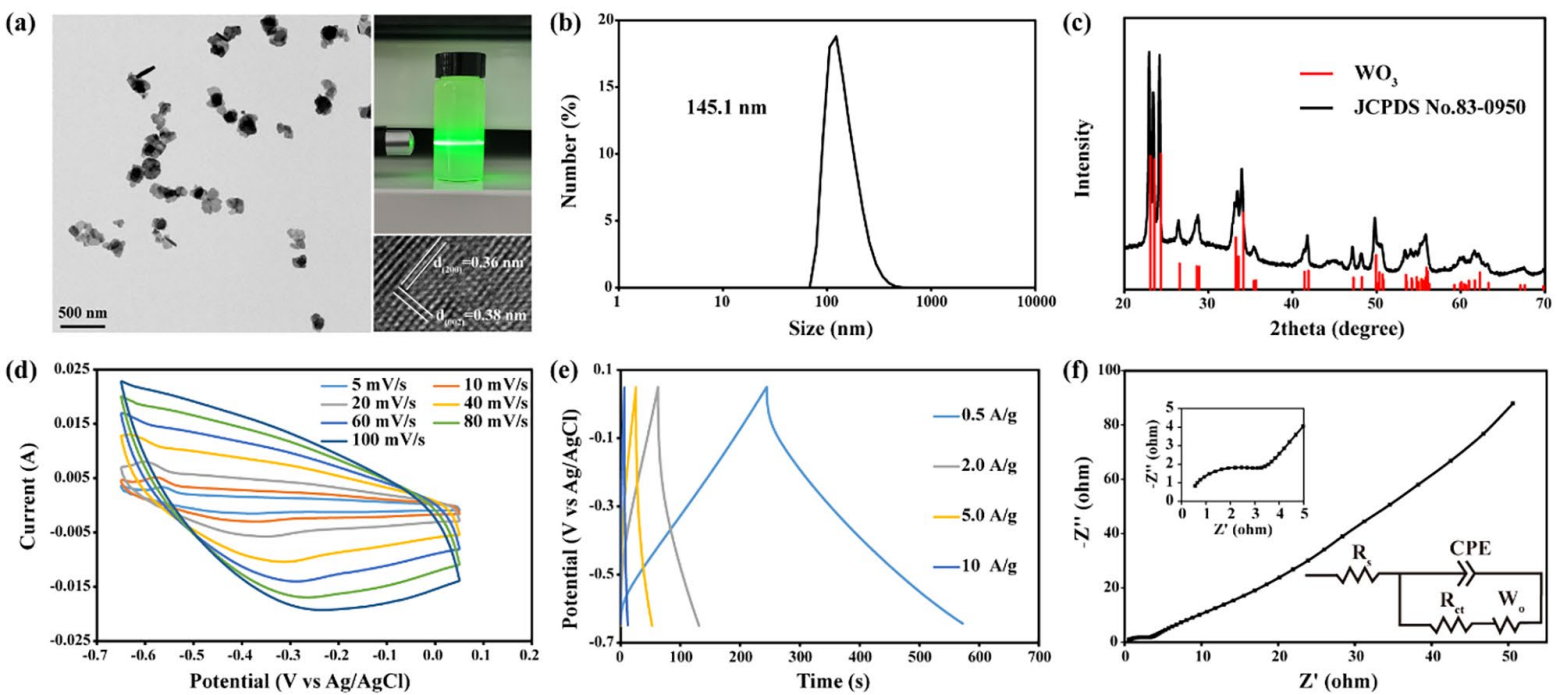
Characterization of WO_3_ nanocapacitors. **(a)** TEM, HRTEM and aqueous solutions images of WO_3_ nanocapacitors. **(b)** Hydrodynamic size of WO_3_ nanocapacitors. **(c)** X-ray diffraction pattern of WO_3_. **(d)** Cyclic voltammograms of WO_3_ nanocapacitors. **(e)** Galvanostatic charge/discharge curves of WO_3_ nanocapacitors. **(f)** Electrochemical impedance spectroscopy of WO_3_ nanocapacitors

WO_3_ nanocapacitors containing oxygen vacancies (WO_3-x_) were synthesized by the hydrothermal reaction between hydrochloric acid, Na_2_WO_4_·2H_2_O, sodium citrate, and glucose [30]. To eliminate vacancies, the as-prepared WO_3-x_ was calcined at 500 ℃ for 10 h. After heat treatment, pale yellow WO_3_ nanocapacitors were obtained. Transmission electron microscopy (TEM) showed that the size of WO_3_ was ~ 100 nm (Fig. 2a). The lattice distance (d_(200)_ and d_(002)_) in the high-resolution TEM (HRTEM) image (Fig. 2a) showed that the exposed crystal face was the (010) face. The hydrodynamic size of WO_3_ measured by dynamic light scattering (DLS) was 145.1 nm (Fig. 2b), presenting the favourable water solubility of WO_3_. The X-ray diffraction (XRD) peak confirmed the successful synthesis of WO_3_ (Fig. 2c). The ultraviolet‒visible (UV‒Vis) spectrum revealed strong absorbance of WO_3_ at approximately 400 nm (Figure S1).

### Electrochemical measurements

After the successful preparation of WO_3_ nanocapacitors, their electrochemical performance was investigated. The redox peaks in cyclic voltammograms (CV) fade away as the scanning speed increased, implying that WO_3_ nanocapacitors were pseudocapacitors (Fig. 2d). The shape of the galvanostatic charge/discharge (GCD) curves and electrochemical impedance spectroscopy (EIS) further favoured that WO_3_ was a pseudocapacitor (Fig. 2e and f), consistent with previous studies [31]. Using the GCD curves, the specific capacitance was calculated to be 236.5 F/g, 196.1 F/g, 191.8 F/g, 80.3 F/g at current densities of 0.5 A/g, 2.0 A/g, 5.0 A/g and 10.0 A/g, respectively. The cycling performance of WO_3_ was also tested. As shown in Figure S2, after 5 thousand charge and discharge cycles at a current density of 5.0 A/g, the capacitance of WO_3_ remained stable. All the electrochemical data concluded that WO_3_ nanocapacitors had satisfying pseudocapacitance performance.

### Regulating electron transportation in the process of radiolysis

**Fig. 3:**
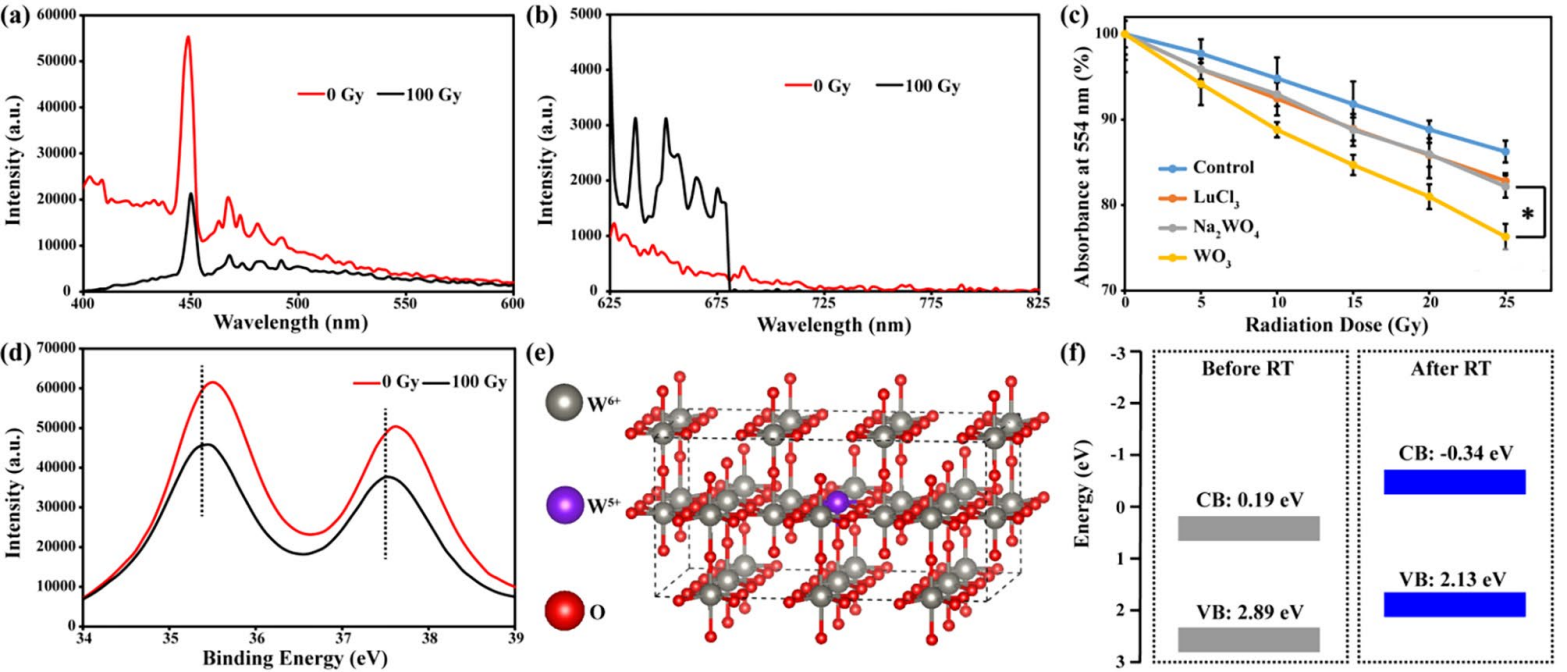
Characterization of the interaction between WO_3_ nanocapacitors and X-ray. **(a)** and **(b)** photoluminescence spectrum of WO_3_ nanocapacitors before and after irradiation of 100 Gy X-rays, respectively. **(c)** The yield of ·OH in solutions upon irradiation of 0 Gy, 5 Gy, 10 Gy, 15 Gy, 20 and 25 Gy X-rays (n = 6, mean ± s.d., asterisk indicates P < 0.05 according to Student’s two-tailed t test). **(d)** XPS spectra of WO_3_ nanocapacitors before and after irradiation of 100 Gy X-rays. **(e)** Simulated WO_3_ nanocapacitors lattice after accepting exogenous electrons. **(f)** Simulated conduction band and valence band before and after accepting exogenous electrons

As radiolysis could generate massive free electrons, these electrons would be partly absorbed by WO_3_ nanocapacitors due to their pseudocapacitance effect. After irradiation with X-rays (100 Gy, 6 MeV), the photoluminescence (PL) spectrum of WO_3_ nanocapacitors solutions (Fig. 3a and b) showed increased longwave fluorescence (625 nm ~ 675 nm) and decreased shortwave fluorescence (400 nm ~ 500 nm). This observation could be attributed to the changed energy band of WO_3_ nanocapacitors induced by excess electrons, which further supported the experimental hypothesis [32, 33].

Radiotherapy utilizes reactive oxygen species, especially ·OH generation pathways for cancer treatment [6, 34]. The yield of ·OH in solutions was measured through the degradation of rhodamine B (RhB). As shown in Fig. 3c, the WO_3_ group could induce more ·OH than the control groups, such as the LuCl_3_ group and Na_2_WO_4_ group. The X-rays could decompose water into ·OH and electrons, while ·OH and electrons could neutralize each other by recombination. However, WO_3_ could function as a pseudocapacitor to absorb electrons, resulting in reduced consumption of ·OH. While LuCl_3_ and Na_2_WO_4_ existed in solution as ions, they cannot act as capacitors. These data also indicated that hexavalent W atoms made little contribution to the absorption of electrons because the yield of ·OH in the Na_2_WO_4_ (Z = 74) group was similar to that in the LuCl_3_ (Z = 71) group. We further investigated whether the electrons could be stored in WO_3_ nanocapacitors. As shown in Fig. 3d, after X-ray irradiation, the binding energy of the W 4f orbital declined. Finally, a simulated calculation was conducted to assess the feasibility of regulating electron transportation. As shown in Fig. 3e, we set up a lattice containing W^6+^ and W^5+^ to simulate WO_3_ nanocapacitors after RT. It was found that before RT, the conduction band of WO_3_ was 0.19 eV; hence, free electrons in solutions (~-2.87 eV) could easily enter the lattice [35]. After receiving electrons, the conduction band of WO_3_ was − 0.34 eV, which could reduce NAD^+^ to NADH (NAD^+^/NADH~-0.32 eV). Both the experimental data and simulated calculation corroborated the feasibility of regulating electron transportation using WO_3_ nanocapacitors.

### In vitro cell experiments

**Fig. 4:**
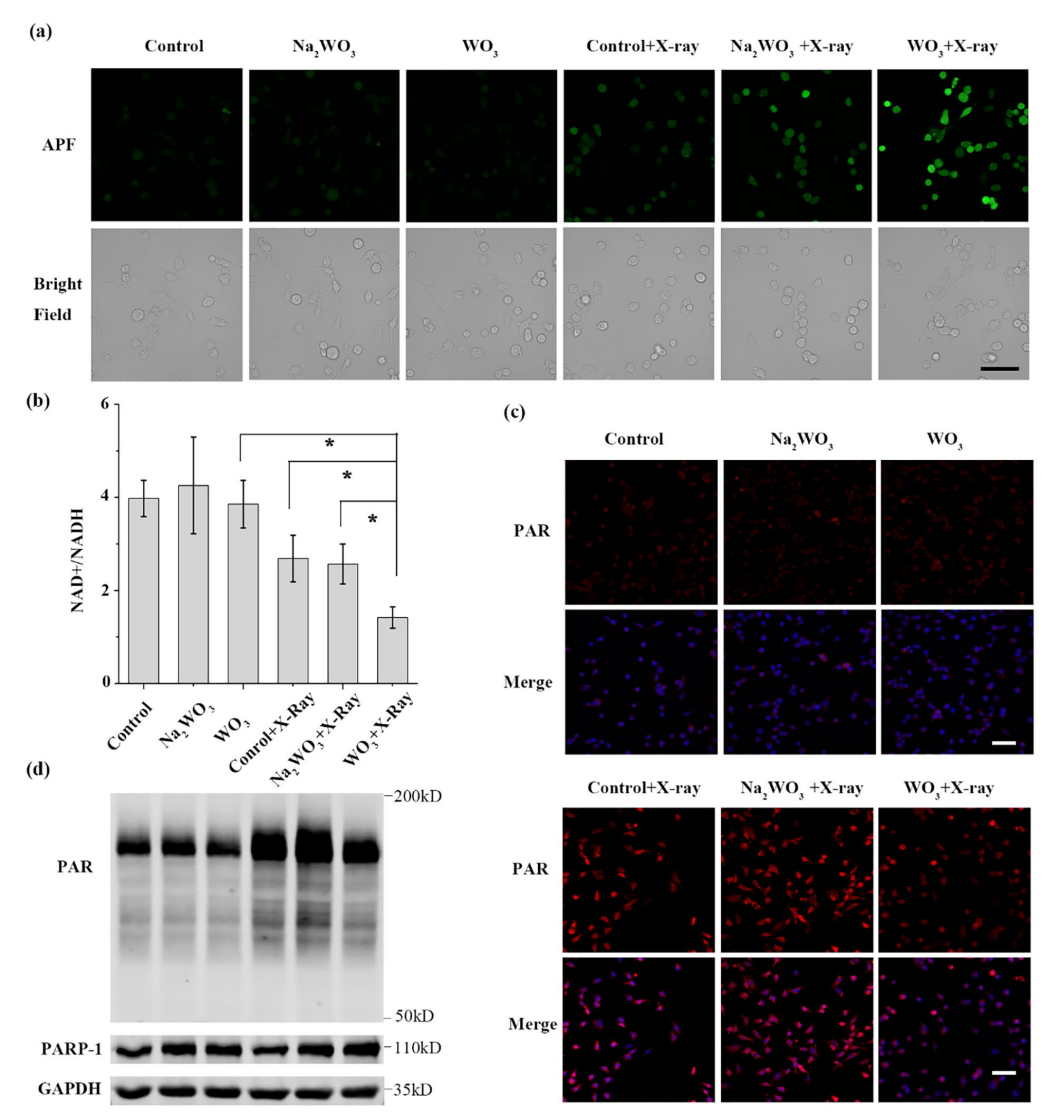
In vitro cell experiments. **(a)** The yield of •OH (measured by APF probe). Scale bar: 50 μm. **(b)** The ration of NAD^+^/NADH (n = 4, mean ± s.d., asterisk indicates P < 0.05 according to Student’s two-tailed t test). **(c)** Immunofluorescence staining of intracellular PAR. Scale bar: 50 μm. **(d)** The content of PARP-1 and PAR.

After the experiments in solutions, in vitro experiments using Lewis cells (mouse lung cancer) were conducted. Firstly, we investigated the stability of WO_3_ nanocapacitors in RPMI-1640 medium. Nonsignificant difference was detected in terms of hydrodynamic size (Figure S3) at 1-, 5- and 10-day period, respectively. In addition, few W atoms were released from WO_3_ nanocapacitors as shown in Figure S4. These data showed that WO_3_ nanocapacitors could remain stable in physiological solutions. Then, the cytotoxicity of WO_3_ nanocapacitors was evaluated via CCK-8 assays. As shown in Figure S5, the viability of Lewis cells and normal cells (RAW264.7 and H9C2 cells) was more than 80%, even at a concentration of 400 µg/mL WO_3_ nanocapacitors, and 20 µg/mL WO_3_ nanocapacitors was used for the following cell experiments. Na_2_WO_3_·2H_2_O (28.5 µg/mL) was also used as a control to balance the possible influence of W on radiation effects. Second, the intracellular content of •OH was detected. As shown in Fig. 4a, compared with the X-ray group and Na_2_WO_3_ + X-ray group, the WO_3_ + X-ray group possessed a higher level of •OH, mainly attributed to the interference of the recombination between electrons and •OH.

In the process of RT, •OH attacks DNA molecules to cause SSBs and DSBs [36]. Although the amount of DNA SSBs is dozens of times that of DSBs, DNA DSBs are accountable for the majority of cancer cell deaths [19]. When DNA SSBs are not repaired in a timely manner, they will convert to fatal DSBs at replication forks [37]. The PARP-1-mediated signalling pathway is one of the most important DDR pathways responsible for DNA SSB repair. In the DNA damage repair process, the activation of PARP-1 occurs after its N-terminal zinc finger DNA binding domain recognizes and interacts with DNA SSBs. Subsequently, PARP-1 utilizes NAD^+^ as a substrate to catalyse poly (ADP-ribose) (PAR) polymerization [37, 38]. The C-terminal domain then transfers PAR polymers to both itself and other neighbouring histones, which leads to the recruitment of various downstream proteins that participate in DNA repair [39]. Undoubtedly, NAD^+^ is the essential substrate for PARP-1 enzymes. Therefore, reducing the intracellular NAD^+^ content will restrain DNA SSB repair mediated by the PARP-1 signalling pathway and increase the number of irreparable DNA SSBs as well as the likelihood of DNA DSBs upon irradiation. As mentioned above, we found that WO_3_ could increase the cellular content of •OH and was expected to decrease the content of NAD^+^ upon irradiation. Hence, the NAD^+^ consumption mediated by WO_3_ nanocapacitors upon irradiation was expected to decrease the expression of PAR as well as the DNA SSB repair efficiency via the PARP-1 signalling pathway and induce more DSBs [40]. Subsequently, the intracellular NAD^+^ content was detected to assess whether the released electrons can reduce intracellular NAD^+^. After radiation, the ratio of NAD^+^/NADH in cells obviously decreased in the presence of WO_3_ nanocapacitors (Fig. 4b), indicating intracellular NAD^+^ reduction, consistent with the results from the simulated calculation. Certainly, the decreased NAD^+^ content would affect the expression of PAR protein.

**Fig. 5:**
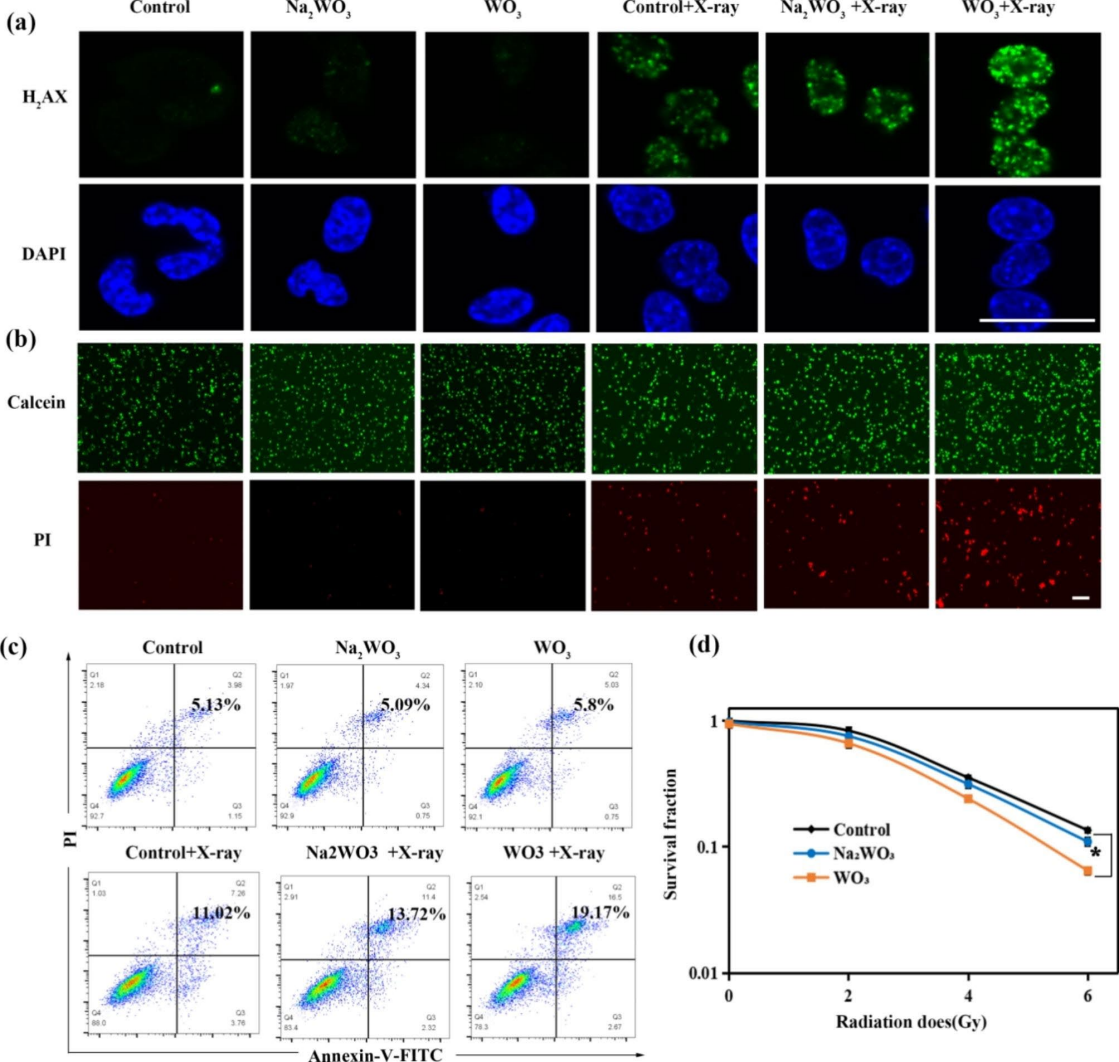
In vitro cell experiments. **(a)** DNA DSBs (measured by γ-H_2_AX staining). Scale bar: 50 μm. **(b)** The Staining of live(green) and dead(red) cells. Scale bar: 50 μm. **(c)** Apoptotic analysis measured by flow cytometry. **(d)** Cell survival assay using colony formation evaluation (n = 3, mean ± s.d., asterisk indicates P < 0.05 according to Student’s two-tailed t test)

To further visualize and quantify the impacts of WO_3_ nanocapacitors on PAR, immunofluorescence staining of intracellular PAR was conducted. As shown in Fig. 4c, all the radiotherapy groups exhibited higher fluorescence intensity than the control group due to the activation of the PARP-1-mediated repair pathway. Meanwhile, the WO_3_ + X-ray group showed the mildest fluorescence intensity among the radiotherapy groups, indicating inhibited expression of PAR compared with the other two groups. Additionally, western blot analysis further proved the lower expression of PAR in the presence of WO_3_ nanocapacitors upon irradiation (Fig. 4d). Both of these results demonstrated the decreased expression of PAR mediated by the reduced intracellular NAD^+^ content, which further downregulated the activity of the PARP-1 signalling pathway. Consequently, more DNA DSBs were detected in the WO_3_ + X-ray groups (Fig. 5a). In line with these findings, the WO_3_ + X-ray group induced the highest apoptotic rate (Fig. 5d and c) and the lowest colony formation rate (Fig. 5d and Figure S6). Taken together, the in vitro experiments validated that WO_3_ nanocapacitors could regulate electron transportation to improve the effect of RT by increasing the yield of •OH and inhibiting DNA damage repair.

### In vivo animal experiments

**Fig. 6:**
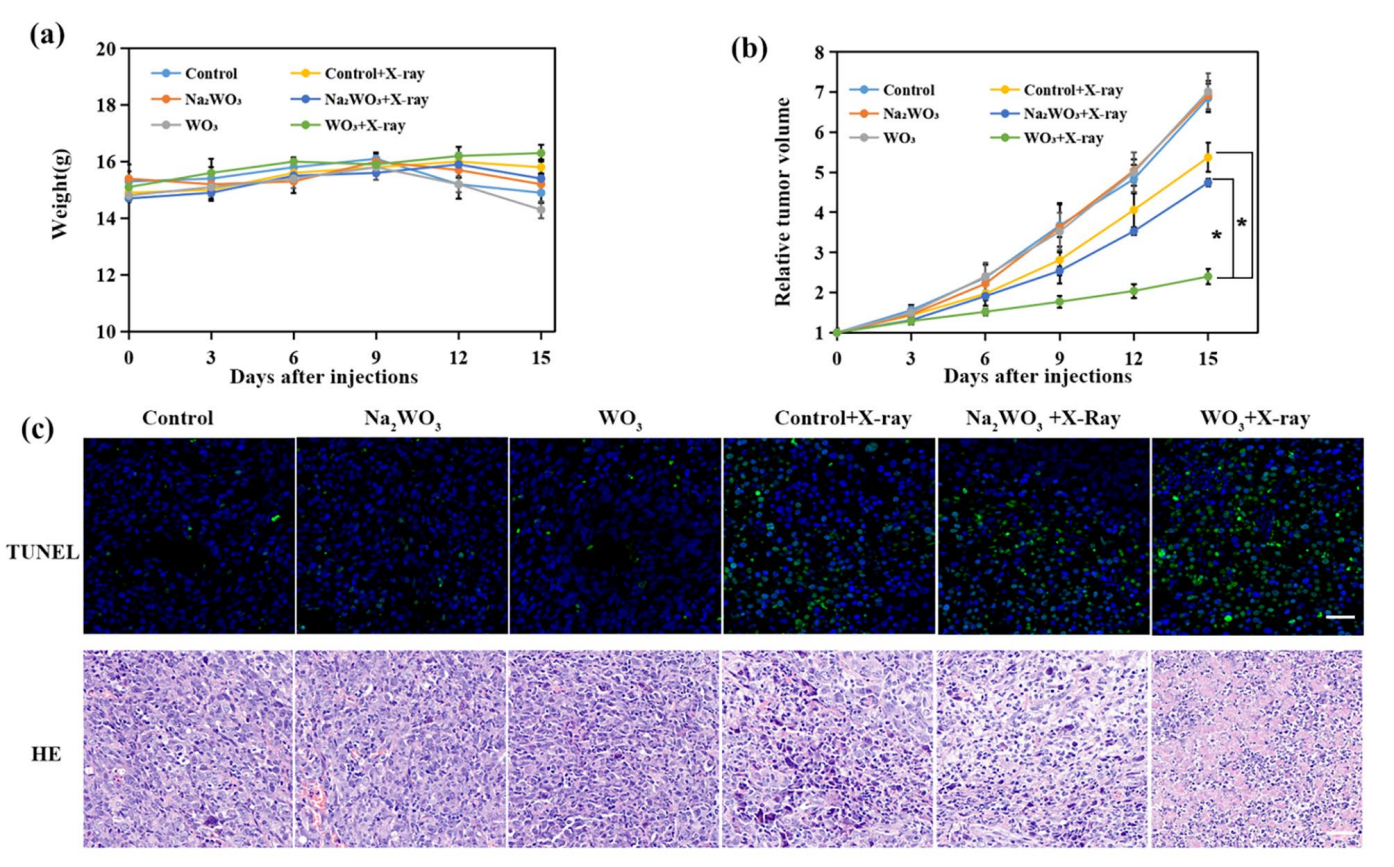
In vivo experiments. **(a)** The change of weights of Lewis tumor-xenografted mice before and after irradiation of 6 Gy X-rays (n = 6, mean ± s.d.). **(b)** Relative tumor volume after intratumoral injection of PBS (10 µL), Na_2_WO_3_ and WO_3_ nanocapacitors (1 mg, 10 µL) with or without irradiation of X-rays (6 Gy, n = 6, mean ± s.d., asterisk indicates P < 0.05 according to Student’s two-tailed t test). **(c)** TUNEL staining and H&E staining of tumor sections. Scale bar: 50 μm

The radiosensitizing effect of WO_3_ nanocapacitors was further investigated using in vivo models. First, the in vivo biocompatibility of WO_3_ nanocapacitors was tested using Kunming mice. The blood half-life of WO_3_ nanocapacitors was approximately one hour (Figure S7), and it was mainly distributed in the liver and spleen after intravenous injection (Figure S8). In line with the in vitro results, no obvious difference between the WO_3_ group and the control group was observed in terms of body weight (Figure S9), haematological index (Figure S10), and histological examination of major organs (Figure S11), implying the favourable biocompatibility of WO_3_ nanocapacitors. Then, tumour-bearing mouse models were established by subcutaneous inoculation of Lewis cells. After intratumoral administration of WO_3_ solutions, the tumours were irradiated with 6 Gy of X-rays. The body weight and tumour size were recorded every third day for 15 days. The weight of the WO_3_ + X-ray group increased slightly, while the other groups showed varying degrees of weight loss (Fig. 6a). The WO_3_ + X-ray group showed the most significant tumour growth retardation compared with the other groups (Fig. 6b and S12). Based on H&E histological staining and terminal deoxynucleotidyl transferase-mediated nick end labelling (TUNEL) assays of tumours, the WO_3_ + X-ray group presented the best therapeutic effect (Fig. 6c). Collectively, the in vivo experimental data validated the feasibility of WO_3_ nanocapacitors-mediated electron-regulated radiosensitization.

## Conclusion

In summary, this study provided a proof of concept of a novel radiosensitization approach based on WO_3_ nanocapacitors by regulating electron transportation in situ. During radiolysis, WO_3_ nanocapacitors absorbed electrons to prevent recombination between electrons and ·OH, contributing to a high cellular level of •OH. After radiolysis, the absorbed electrons were released to react with the PARP-1 substrate NAD^+^. As a result, PARP-1-mediated DNA SSB repair was inhibited, which created more complicated DNA damage upon irradiation. The in vitro and in vivo experiments provided solid evidence for this hypothesis. Collectively, this strategy of nanocapacitor-based radiosensitization improves the radiotherapeutic effects by increasing the utilization of radiolytic electrons and ·OH, warranting further validation in multiple tumour models and preclinical experiments.

## Electronic supplementary material

Below is the link to the electronic supplementary material.

Supplementary Material 1

## Data Availability

All study data are included in this article. Received: ((will be filled in by the editorial staff)). Revised: ((will be filled in by the editorial staff)). Published online: ((will be filled in by the editorial staff))
