# Supplementary material for: Regulating electron transportation by tungsten oxide nanocapacitors for enhanced radiation therapy
Source: J Nanobiotechnology. 2023 Jun 29;21:205. doi: 10.1186/s12951-023-01962-8 (PMC10308650; doi:10.1186/s12951-023-01962-8)
Supplement: Supplementary file 1 — Supplementary Material 1 [file 12951_2023_1962_MOESM1_ESM.docx]

**Supporting Information**

**Regulating Electron Transportation by Tungsten Oxide Nanocapacitors for Enhanced Radiation Therapy**

Hongbo Gao^1†^, Li Sun^1†^, Dalong Ni^2^, Libo Zhang^3^, Han Wang^2^, Wenbo Bu^4^, Jinjin Li^5^, Qianwen Shen^1^, Ya Wang^4^, Yanyan Liu^4^**, Xiangpeng Zheng^1^*

^1^ Department of Radiation Oncology, Shanghai Huadong Hospital, Fudan University, Shanghai,200040 ,China.

^2^ Department of Orthopaedics, Shanghai Key Laboratory for Prevention and Treatment of Bone and Joint Diseases, Shanghai Institute of Traumatology and Orthopaedics, Ruijin Hospital, Shanghai Jiao Tong University School of Medicine, Shanghai 200025, China.

^3^ Department of Radiology, the First Affiliated Hospital of Soochow University, Suzhou 215006, China.

^4^ Department of Material Science and State Key Laboratory of Molecular Engineering of Polymers, Fudan University, Shanghai, 200433 , China.

^5^ Shanghai Key Laboratory of Green Chemistry and Chemical Processes, School of Chemistry and Molecular Engineering, East China Normal University, Shanghai 200062, China.

^*^  Corresponding author at: Department of Radiation Oncology, Huadong Hospital Affiliated to Fudan University, Shanghai 200040, China.

^**^ Corresponding author at: Department of Materials Science, Fudan University, Shanghai 200433, China.

*E-mail addresses*: zhengxp@fudan.edu.cn (X. Zheng), liuyyan@fudan.edu.cn (Y. Liu)

^†^ These authors contributed equally to this work.

Keywords: tungsten oxide, cancer, radiotherapy, nanotechnology, pseudocapacitor

**Supplementary Figures and Tables**


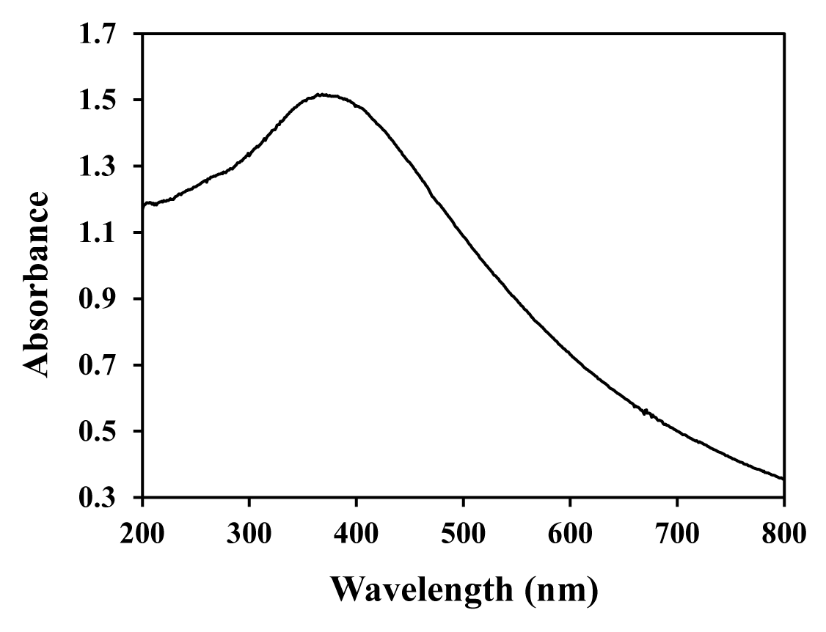


**Figure S1.** UV-Vis spectra of WO_3_ nanocapacitors.


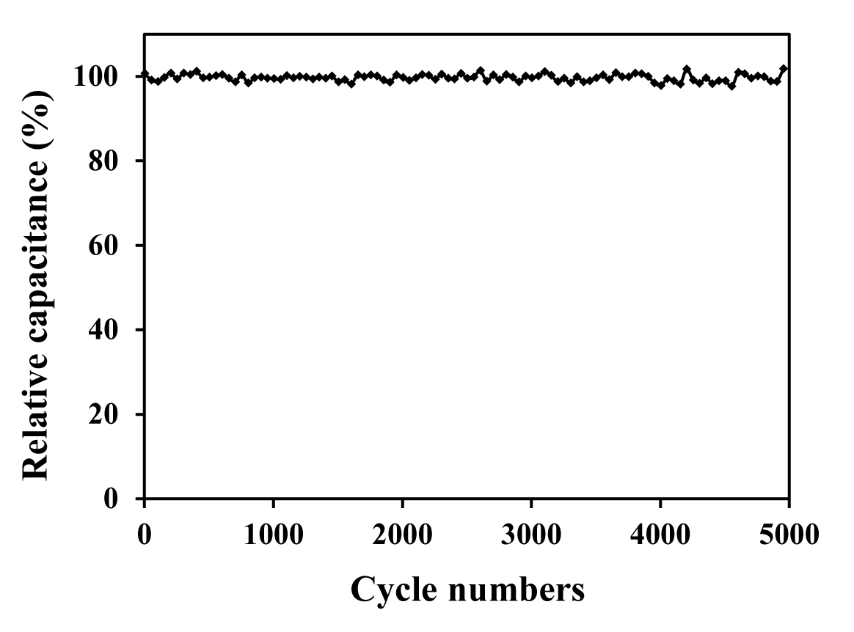


**Figure S2.** Cycling performance of WO_3_ nanocapacitors at a current density of 5 A/g.


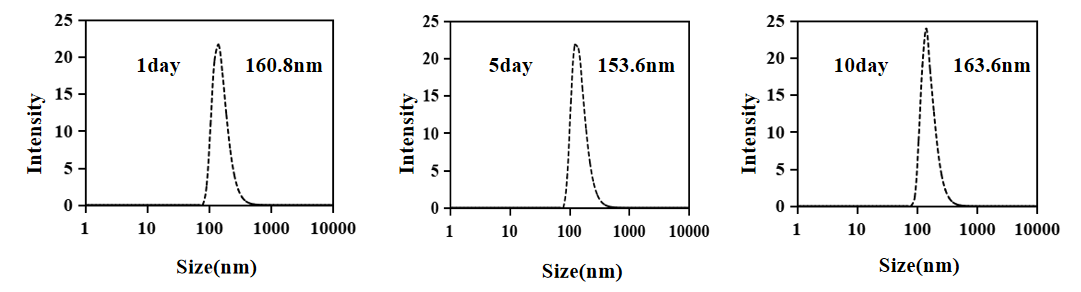


**Figure S3:** Hydrodynamic size of WO_3_ nanocapacitors dispersed in RPMI-1640 medium at 1 day, 5 day and 10 day.


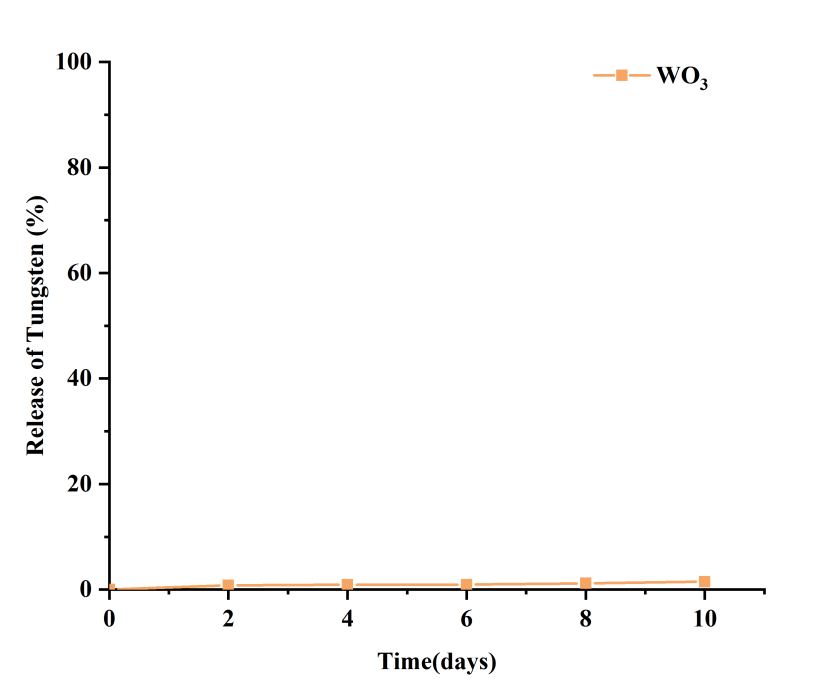


**Figure S4:** The release of tungsten when WO3 nanocapacitors dispersed in RPMI-1640 medium at 2 day, 4 day, 6 day, 8 day, 10 day.


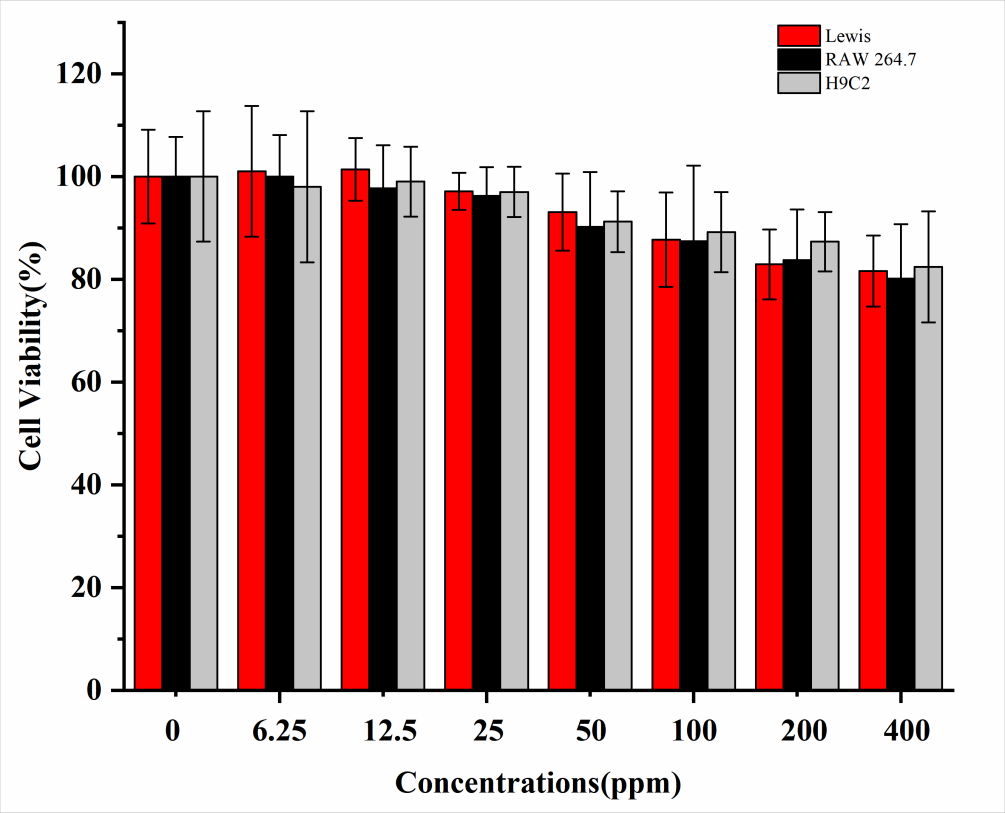


**Figure S5.** Cell viability of WO_3_ nanocapacitors with different concentrations (n=6).


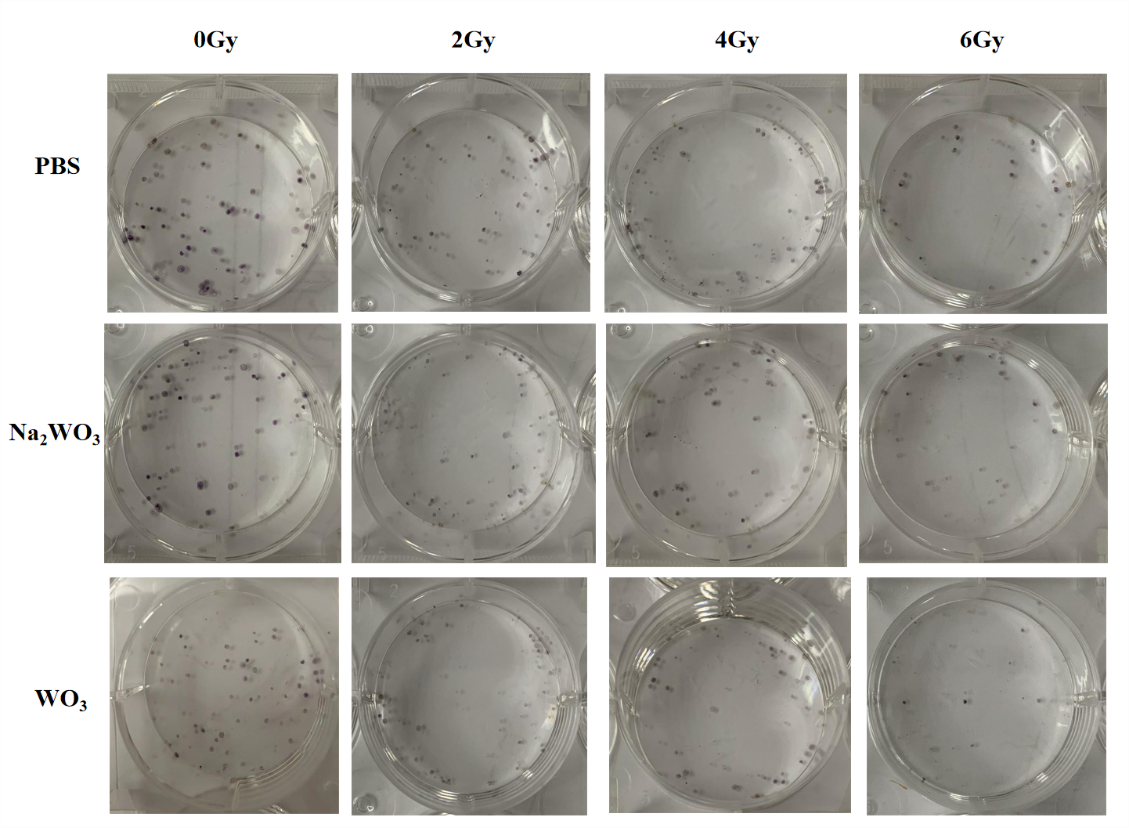


**Figure 6.** The pictures of clone formation assay


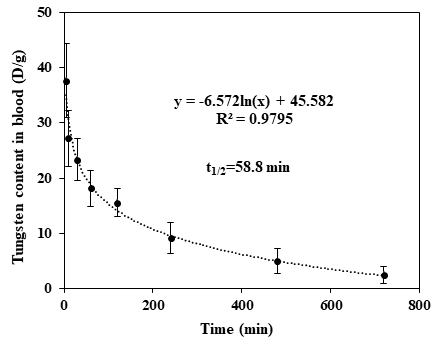


**Figure S7.** In vivo blood terminal half-life (t_1/2_) of WO_3_ nanocapacitors after intravenous injection (50 mg/kg, n=3).


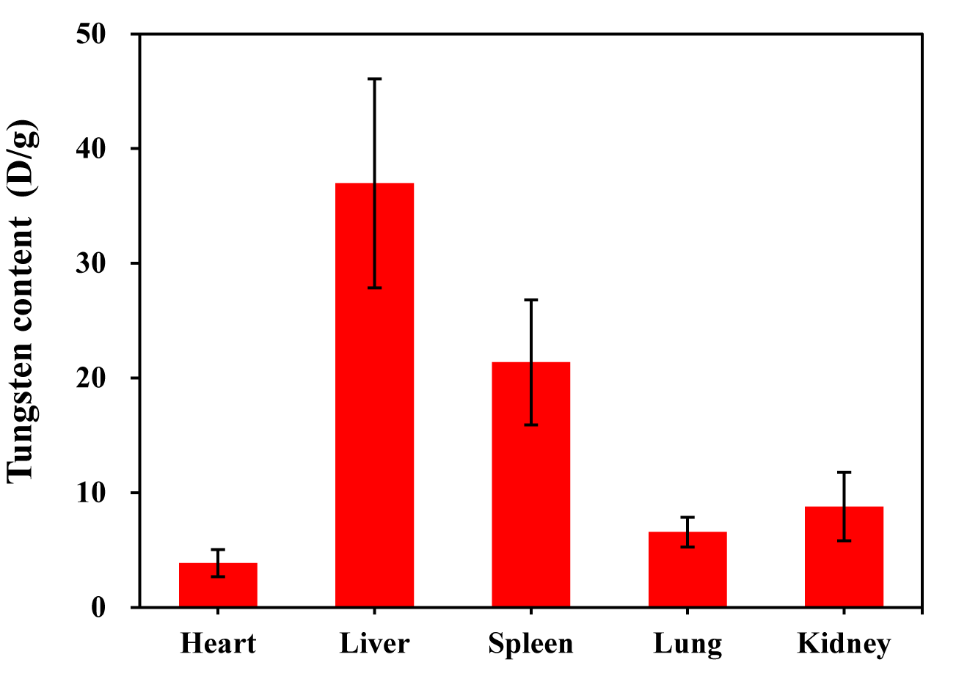


**Figure S8.** In vivo tissue biodistribution in major organs (heart, liver, spleen, lung, kidney) 24 hours after intravenous injection of WO_3_ nanocapacitors (50 mg/kg, n=3).


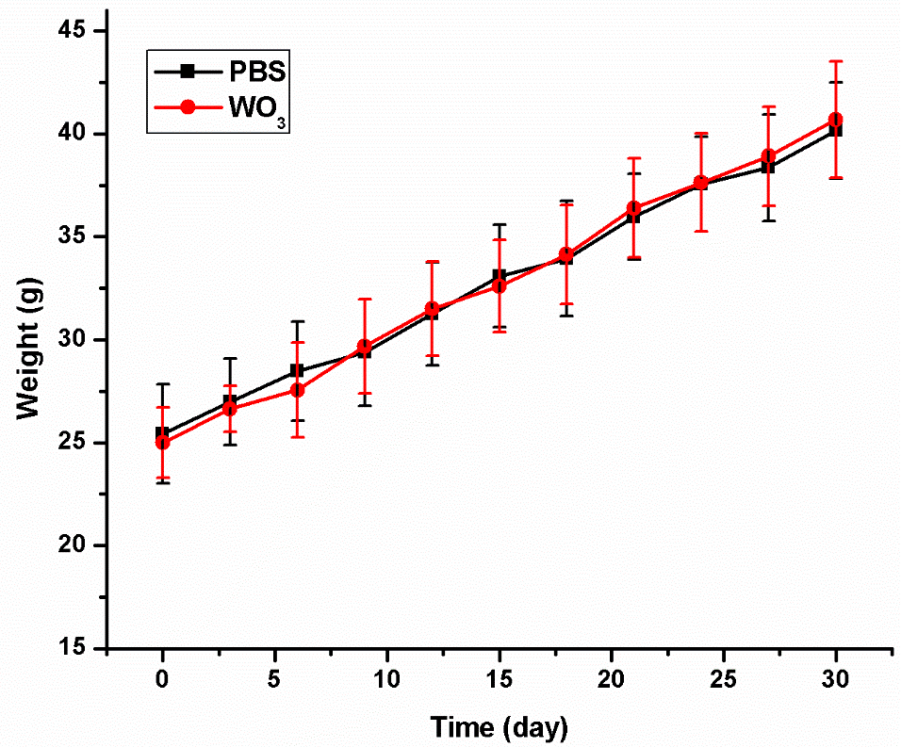


**Figure S9.** The change of weights of Kunming mice after intravenous injection of WO_3_ nanocapacitors (50 mg/kg, n=5).


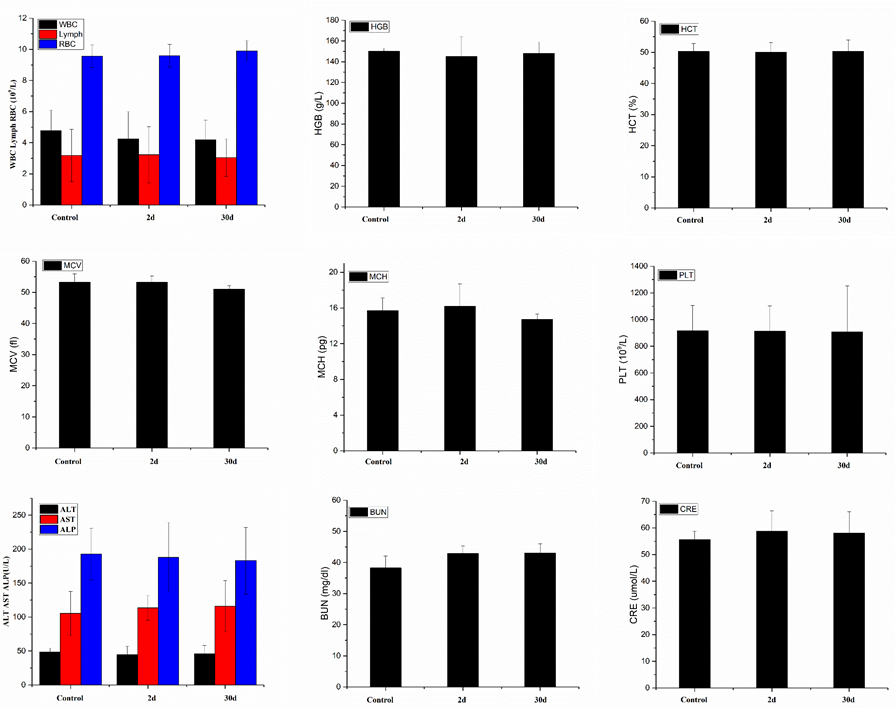
**Figure S10.** Blood routine and biochemical indexes in vivo 2 days and 30 days after intravenous injection of WO_3_ nanocapacitors (50mg/kg, n=5).

**
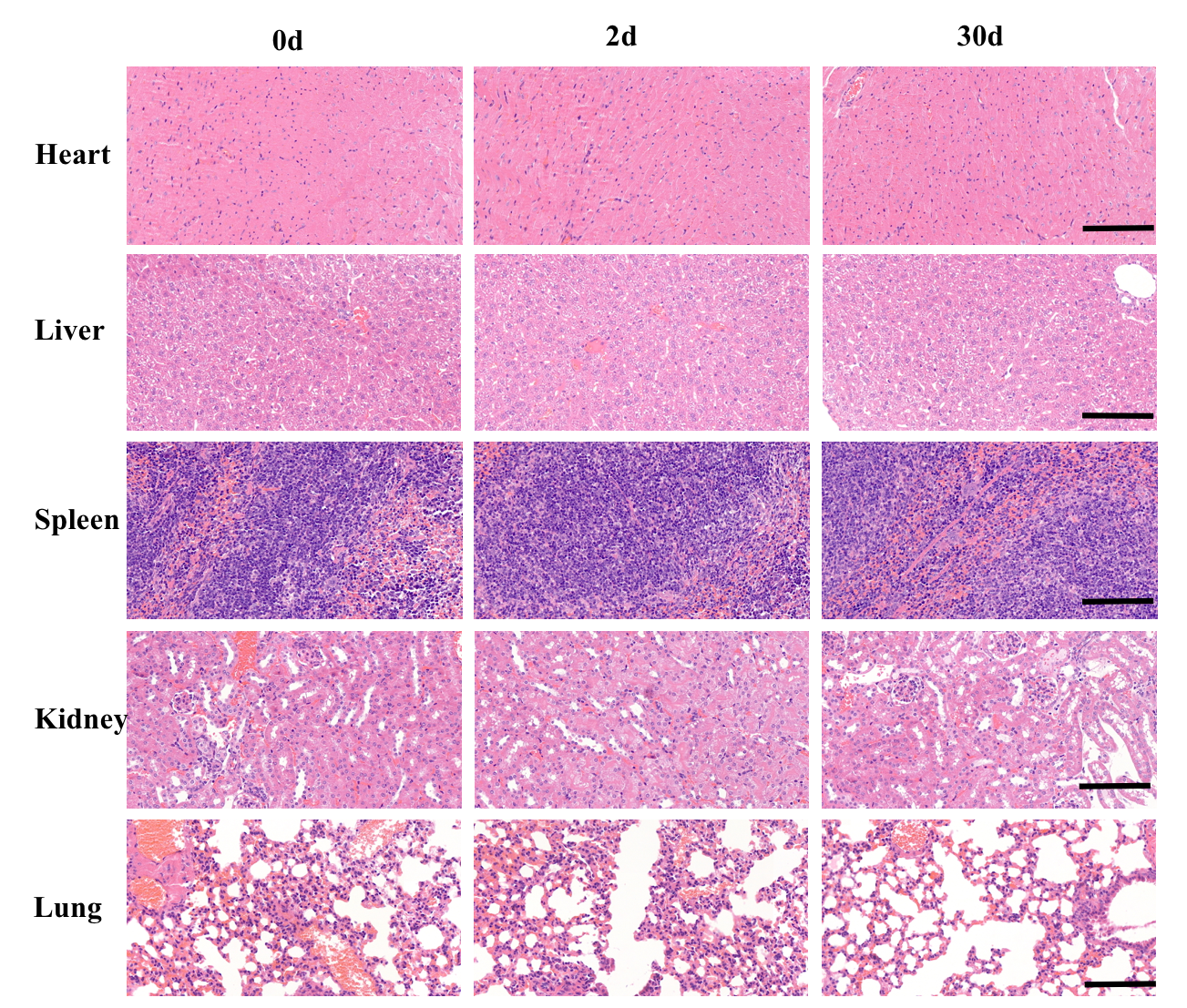
Figure S11.** In vivo H&E staining of major organs (heart, liver, spleen, lung, kidney) 2 days and 30 days post intravenous injection of WO_3_ nanocapacitors (50 mg/kg). Scale bar: 50 μm.


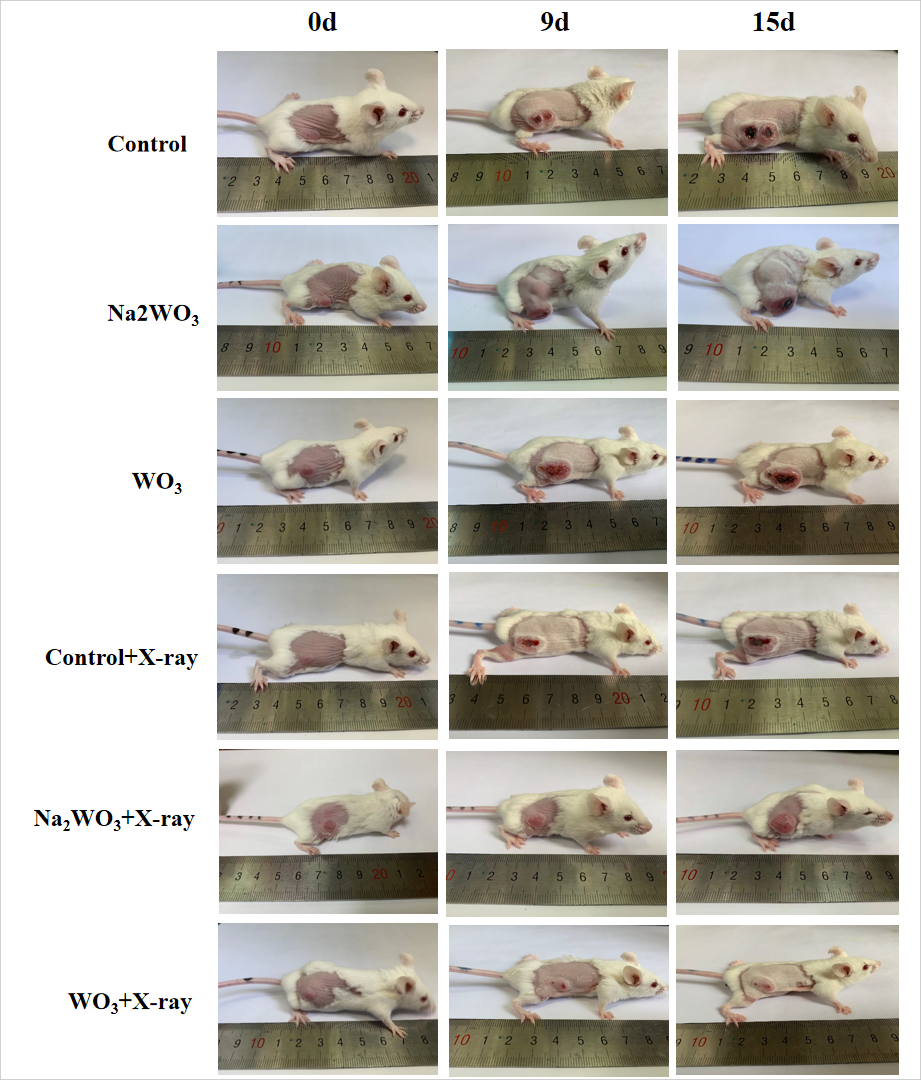


**Figure S12.** Photographs of tumor-xenografted mice after intratumor injection of PBS (10 μL), Na_2_WO_3_ (1.4mg ,10 μL) and WO_3_ nanocapacitors (1 mg, 10 μL) with or without irradiation of 6 Gy X-rays.
